# Prevalence of Injuries Among Medicaid Enrolled Infants Prior to Child Abuse and Neglect

**DOI:** 10.1177/10775595211031651

**Published:** 2021-07-28

**Authors:** Meghan E. Shanahan, Anna E. Austin, Molly C. Berkoff

**Affiliations:** 1 Department of Maternal and Child Health, Gillings School of Global Public Health, 41474 University of North Carolina, Chapel Hill, NC, USA; 2 Injury Prevention Research Center, University of North Carolina, Chapel Hill, NC, USA; 3 Department of Pediatrics, School of Medicine, 2331 University of North Carolina, Chapel Hill, NC, USA

**Keywords:** child maltreatment, prevention, injury prevention

## Abstract

Prior research has identified common injuries among children who experience child maltreatment; however, most of this work has focused on inpatient settings and has excluded many cases of neglect. This study examines the prevalence of injuries that occur prior to a diagnosis of child maltreatment, as well as the proportion of children with well-child visits prior to the injury and child maltreatment diagnosis. Based on a secondary analysis of Medicaid data from four states, we found that among infants with 12 months of continuous enrollment (*N* = 4817), 30.6% of those diagnosed with maltreatment were previously diagnosed with an injury. Among infants diagnosed with child maltreatment, 88.4% had at least one well-child visit prior to the maltreatment diagnosis. Among children with a maltreatment diagnosis and a prior injury, 84% had at least one well-child visit preceding the injury. These results indicate that most children had at least one well-child visit prior to being diagnosed with child maltreatment or an injury, indicating opportunities for prevention.

## Introduction

Child maltreatment, also referred to as child abuse and neglect, is a significant public health problem. According to child protective services (CPS) data, approximately 678,000 children experienced abuse and neglect in 2018 (US Department of Health and Human Services, 2020), a number which underestimates the true extent of child maltreatment in the United States (Sedlak et al., 2010). In addition to the individual impact of abuse and neglect, there is a large economic burden. The cost of treating the negative sequelae associated with abuse, and neglect, and providing services to families through CPS is estimated to be $830,928 per victim (Peterson et al., 2018). Further, between 20–42% of children who are maltreated experience recurrent episodes (White, 2015) and, of concern, risk of mortality increases with each occurrence of child maltreatment (Deans et al., 2013; Oral et al., 2008). Therefore, it is important to identify children who may be at risk for maltreatment, or who have experienced relatively minor injuries as a result of maltreatment, as early as possible to intervene before serious physical or psychological injury occurs.

Given their specific training in child health and well-being, as well as the frequency in which they interact with children and their families (American Academy of Pediatrics & Section on Radiology, 2009; Flaherty et al., 2010), pediatricians may be uniquely positioned to identify children who have experienced maltreatment (Christian, 2015). However, research demonstrates that instances of abuse and neglect are often not identified by pediatricians (Hymel et al., 2018; Jenny et al., 1999; Lane et al., 2002). Notably, prior work suggests that certain injuries are more common among children who are eventually diagnosed with maltreatment (Lindberg et al., 2015; Puls et al., 2018; Sheets et al., 2013; Thackeray et al., 2016). Specifically, one study examined infants less than 12 months of age with definite abuse and found that more than one-fourth (28%) had a previous injury suggestive of maltreatment (i.e., a sentinel injury). The most common injury was a bruise (80%), followed by an intraoral injury (11%), and a fracture (7%; Sheets et al., 2013). In addition, prior injuries were common in infants with definite abuse but were rare in infants evaluated by a hospital-based Child Protection Team and found not to have been abused (Sheets et al., 2013). Another study examined physical abuse diagnoses among children less than 24 months of age with injuries potentially indicative of abuse and determined that specific injury diagnoses, such as rib fractures (56%), abdominal trauma (25%), and subconjunctival hemorrhage (i.e., broken blood vessel in the eye; 9%), were relatively common among children with abuse diagnoses (Lindberg et al., 2015). Thackeray et al. (2016) determined that children less than 12 months of age with an abuse diagnosis or a skeletal survey, which often indicates provider concern for abuse, contusions (28%), fractures (27%), open wounds (17%), and superficial injuries (12%) were relatively common. In a study of inpatient pediatric hospitalizations, children who were diagnosed with physical abuse were more likely to have previous diagnoses of fractures, head injuries, and symptoms concerning for abusive head trauma than children with unintentional injuries (Puls et al., 2018).

These prior studies point to the potential for certain injuries among young children to serve as an indicator of possible abuse and harm to the child and a signal for further evaluation and assessment by the medical team. However, none of these studies included specific neglect diagnoses distinct from abuse when identifying children who experienced maltreatment. This is important as neglect may result in injuries due to improper supervision or unsafe environments (Damashek et al., 2014; Notrica et al., 2020) and is more common than physical abuse (US Department of Health and Human Services, 2020). Further, two of the studies only utilized data regarding pediatric hospital encounters (i.e., inpatient, emergency department, ambulatory surgery, and observation data) to identify cases of both abuse and minor injuries (Lindberg et al., 2015; Puls et al., 2018), thereby missing both abuse and injury diagnoses made in outpatient settings, which may be both more common and less severe than those diagnosed in an inpatient setting. An additional study only included children who were diagnosed with abuse by an inpatient hospital-based Child Protection Team but reviewed all available medical records to identify previous injuries (Sheets et al., 2013). The only study that used data from all medical encounters to identify both injury and abuse diagnoses included injury diagnoses that occurred both before and after the abuse diagnoses (Thackeray et al., 2016). Focusing specifically on injuries that occurred prior to maltreatment is critical to informing primary and secondary maltreatment prevention efforts. Indeed, only two previous studies explicitly examined injuries that occurred prior to the child maltreatment diagnosis (Puls et al., 2018; Sheets et al., 2013).

We aimed to build upon this previous work by using Medicaid claims data for all medical encounters to examine the prevalence of injuries among infants less than 12 months of age prior to a diagnosis of abuse or neglect. Further, to inform potential opportunities to intervene before serious harm occurs, we examined the number of well-child visits among children diagnosed with abuse and neglect, as well as those who experienced both prior injuries and maltreatment.

## Methods

### Data Source

We used data from the 2006–2011 Medicaid Analytic eXtract (MAX) files for California, Georgia, North Carolina, and Texas accessible through the Cecil G. Sheps Center at the University of North Carolina at Chapel Hill. These four states were included in the analysis because they were the only states available under the data use agreement. Each state’s Medicaid agency uses the Medicaid Statistical Information System (MSIS) to submit quarterly eligibility, enrollment, and claims data for all Medicaid and Children’s Health Insurance Program (CHIP) beneficiaries to the Centers for Medicare and Medicaid Services (CMS). The information from the MSIS is then used to create MAX data files to support policy analysis and research. MAX files include beneficiary eligibility and demographic data as well as service utilization, diagnoses, and payment source information for inpatient and outpatient visits.

### Study Population

Our study population included all live born infants who were enrolled in Medicaid or CHIP at birth for 2006–2009 births in California, Georgia, North Carolina, and Texas and 2010 births in Georgia and North Carolina. Additionally, infants needed to have 12 months of continuous enrollment in Medicaid and be diagnosed with child maltreatment prior to age 12 months.

### Measures

*Child maltreatment diagnoses.* We used International Classification of Disease, Ninth Revision, Clinical Modification (ICD-9-CM) codes to identify children who experienced child maltreatment. Consistent with previous research (Puls et al., 2018; Thackeray et al., 2016), we defined child maltreatment as a diagnosis of unspecified child abuse (995.50), child neglect (995.52), child physical abuse (995.54), abusive head trauma (995.55), other child abuse and neglect (995.59), accident due to abandonment or neglect of infants and helpless persons (E904), homicide and injuries intentionally inflicted by other persons (E960-E968), family disruption due to child welfare custody (V61.05), family disruption due to child in foster care or care of non-parental family member (V61.06), and observation and evaluation for suspected abuse and neglect (V71.81). As previously stated, infants who received at least one of these codes in the first 12 months of life were included in the study. If infants had more than one maltreatment diagnosis event, we used the first event in the analyses.

*Injury diagnoses.* Based on extant literature (Lindberg et al., 2015; Thackeray et al., 2016), we used ICD-9-CM codes to identify inpatient and outpatient diagnoses for injuries that occurred prior to the first child maltreatment diagnosis (Table 1). We included diagnosis codes for injuries such as fractures, lacerations, abrasions, and contusions. We also included procedural codes for skeletal surveys, which often indicate that the physician had concerns for occult abusive injuries (Wood et al., 2014). We excluded injury incidents with an accompanying diagnosis, indicating the injury occurred due to an external trauma mechanism (e.g., motor vehicle accident), underlying medical condition, or minor cutaneous injury (e.g., insect bite or splinter), or was coded during an episode of follow-up care (Table 2). As we were interested in injuries that occurred prior to diagnoses of child maltreatment, we also excluded injuries that were diagnosed on the same day as the maltreatment diagnosis. We also excluded injury diagnoses that occurred on the day of birth to ensure we did not include any birth-related injuries.

**Table 1.** Previous Injury Diagnoses and ICD-9-CM Codes.

Previous Injury	ICD-9-CM Codes
Skeletal survey	88.31, 77074
Retinal injury	361, 362
Skull fracture	800–804
Neck or trunk fracture	805–811
Upper-limb fracture	812–819
Lower-limb fracture	820–829
Dislocation	830–839
Sprains	840–848
Intracranial injury	850–854
Internal injury of thorax, abdomen, or pelvis	860–869
Open wound	870–897
Injury to blood vessels	900–904
Superficial injury	910–919
Contusion	920–924
Crushing injury	925–929
Effects of foreign bodies	930–939
Burns	940–949
Injury to nerves or spinal cord	950–957

*Note.* ICD-9-CM = International Classification of Disease, Ninth Revision, Clinical Modification.

**Table 2.** Exclusionary Diagnoses and ICD-9-CM Codes.

Exclusion Criteria	ICD-9-CM Code
Trauma mechanisms for injury	Railway accident (E800-E807)
	Motor vehicle accident (E810-E825)
	Other road vehicle accident (E826-E829)
	Water transport accident (E830-E838)
	Air/space transport accident (E840-E845)
	Vehicle accident not classified (E846-E848)
Underlying medical conditions	Osteogenesis imperfecta and other musculoskeletal anomalies (756)
	Coagulation defect (286, 287, V83.02, 713.2, 776)
	Liver disease (571–573)
Minor cutaneous injuries	Insect bite or splinter (910.4–910.7, 911.4–911.7, 912.4–912.5, 914.4–914.77, 915.4–915.7, 916.4–916.7, 919.7)
Episodes of follow up care	V24, V67

*Note.* ICD-9-CM = International Classification of Disease, Ninth Revision, Clinical Modification.

*Well-child visits.* We used ICD-9-CM codes for routine infant or child check (V202) and newborn health supervision (V20.3) to identify well-child visits that occurred prior to the first injury and prior to the first child maltreatment diagnosis.

### Statistical Analyses

We used descriptive statistics to calculate the prevalence of child maltreatment diagnoses, the prevalence of prior injuries, and the number of well-child visits. We examined the distribution of sex, race, and ethnicity among children with and without previous injuries to determine if there were important differences in demographic characteristics between these groups. All analyses were conducted using SAS 9.4. This study was reviewed and approved by the Institutional Review Board (IRB) at [The University of North Carolina at Chapel Hill].

## Results

Of the 2,170,661 infants born in 2006–2010 with 12 months of continuous Medicaid enrollment, 4817 (0.2%) received a diagnosis of abuse or neglect prior to 1 year of age. The study sample characteristics are included in Table 3. Of infants with a maltreatment diagnosis, 1473 (30.6%) were previously diagnosed with an injury. Of note, the distribution of sex, race, and ethnicity did not differ substantially between children with a previous injury and those without one (Table 3). The average age at the first injury diagnosis was 133 days (4.4 months; std = 95.0). On average, there were 42.6 days (std = 67.2) between the first injury diagnosis and the maltreatment diagnosis.

**Table 3.** Study Sample Characteristics by Presence of Previous Injury, 2006–2010 Births^a^.

	Overall (*N* = 4817)	Previous Injury (*N* = 1473)	No Previous Injury (*N* = 3344)
	*N* (%)	*N* (%)	*N* (%)
Sex			
Male	2673 (55.5)	831 (56.4)	1842 (55.1)
Female	2144 (44.5)	642 (43.6)	1502 (44.9)
Race/ethnicity			
White, non-Hispanic	1725 (35.8)	509 (34.6)	1216 (36.4)
Black, non-Hispanic	1181 (24.5)	378 (25.7)	803 (24.0)
Other/multiple race, non-Hispanic	438 (9.1)	153 (10.4)	285 (8.5)
Hispanic	1473 (30.6)	433 (29.4)	1040 (31.1)

^a^California, Georgia, North Carolina, and Texas Medicaid and Children’s Health Insurance Program enrolled infants; 2010 births for Georgia and North Carolina only.

Among infants with maltreatment diagnoses, the most common prior incidents indicating potential harm were skeletal surveys (38.7%) and contusions (25.4%; Table 4). More than half of the infants with a previous injury (*n* = 760) experienced more than one type of injury prior to being diagnosed with child maltreatment. Specifically, 28.0% experienced two types, 13.7% experienced three types, 6.3% experienced four types, and 3.5% experienced five or more types.

**Table 4.** Types of Previous Injuries Among Children Diagnosed with Child Maltreatment, 2006–2010 Births^a^.

	*N* (%)
Skeletal survey	570 (38.7)
Contusion	374 (25.4)
Skull fracture	291 (19.8)
Intracranial injury	284 (19.3)
Lower-limb fracture	271 (18.4)
Retinal injury	256 (17.4)
Upper-limb fracture	236 (16.0)
Superficial injury	129 (8.8)
Neck or trunk fracture	124 (8.4)
Open wound	82 (5.6)
Effects of foreign body	52 (3.5)
Burns	47 (3.2)
Internal injury of thorax, abdomen, or pelvis	30 (2.0)
Sprains	23 (1.6)
Dislocation	22 (1.5)
Injury to nerves or spinal cord	*
Injury to blood vessels	*
Crushing injury	*

^a^California, Georgia, North Carolina, and Texas Medicaid and Children’s Health Insurance Program enrolled infants; 2010 births for Georgia and North Carolina only.

*Suppressed due to *N* < 10.

Among infants with previous injuries (*N* = 1473), the most common type of maltreatment diagnosis was physical abuse (58.9%), followed by unspecified abuse and or neglect (27.3%), abusive head trauma (14.6%), neglect (12.4%), injury purposely inflicted by other persons (9.4%), and other abuse or neglect (7.9%).

Among infants with a child abuse and neglect diagnosis, 4258 (88.4%) had at least one well-child visit prior to the maltreatment diagnosis. Among children with a maltreatment diagnosis and a prior injury, 1239 (84.0%) had at least one well-child visit prior to the injury diagnosis.

## Discussion

To prevent serious physical and psychological injury resulting from child maltreatment, it is important to identify early indicators of risk for potential harm that may prompt further evaluation and assessment by medical providers. This is not only essential to preventing serious harm, it is also more effective than treating the negative sequelae resulting from trauma and adversity (Kilburn & Karoly, 2008). In a population of infants who were continuously enrolled in Medicaid or CHIP, we found that almost one-third of maltreated infants were diagnosed with an injury prior to the maltreatment diagnosis. We did not find substantial differences in sex, race, or ethnicity between children with previous injuries and those who did not receive an injury diagnoses prior to their maltreatment diagnosis. We identified specific injury diagnoses that were relatively common among maltreated infants and determined that most infants had at least one well-child visit prior to the maltreatment diagnosis, as well as prior to the injury. All these findings point to opportunities for prevention.

Our result demonstrating that injury diagnoses are common among children who experience abuse and neglect is consistent with prior research (Lindberg et al., 2015; Puls et al., 2018; Sheets et al., 2013; Thackeray et al., 2016). Previous studies found that between 13–28% of children who were diagnosed with abuse in an inpatient setting had an earlier diagnosis of at least one minor injury (Puls et al., 2018; Sheets et al., 2013). Our study builds upon this literature by using information from all medical encounters to capture diagnoses of child maltreatment and the injuries that precede them. We also included the specific diagnosis code for neglect, and therefore our study comprised a more broadly defined sample than previous research. These two factors may explain why we found a slightly higher proportion of injuries among children with maltreatment diagnoses than studies that relied solely on hospital data (16.6%; Puls et al., 2018) and did not explicitly include neglect diagnoses (27.5%; Sheets et al., 2013).

Consistent with previous research, we found that contusions were the most common prior injury among children who were later diagnosed with abuse or neglect (Sheets et al., 2013; Thackeray et al., 2016). We also found that prior skeletal surveys were common among children with abuse or neglect diagnoses. We examined the occurrence of prior skeletal surveys because this procedure indicates the physician may have had concerns for potentially abusive injuries (Lindberg et al., 2015; American Academy of Pediatrics & Section on Radiology, 2009). Prior studies have demonstrated the value of skeletal surveys for identifying occult injuries and aiding physicians in recognizing and diagnosing abuse in young children (Christian, 2015; American Academy of Pediatrics & Section on Radiology, 2009). Notably, in our study, over one-third of children diagnosed with abuse or neglect received a skeletal survey during a prior medical encounter but were not concurrently diagnosed with child maltreatment, indicating there may have been earlier concerns for potentially abusive injuries. Overall, our findings illustrate that many children who are diagnosed with maltreatment display indicators of this outcome at previous medical encounters.

Most children in our sample had at least one well-child visit prior to both the injury and maltreatment diagnoses. These visits provide opportunities for physicians to identify children who are at an increased risk of maltreatment, provide support and guidance, and as needed refer families to services that can reduce risk factors and increase protective factors for child abuse and neglect. Ideally, physician offices would implement a universal program, such as Safe Environment for Every Kid (SEEK; Dubowitz et al., 2009; Dubowitz et al., 2012), that screens all families for risk factors for abuse and neglect (e.g., food insecurity, major parental stress, and parental depression) and has in-office support to link families to resources. Screening all families for risk factors reduces stigma (Gross et al., 2020; Sebert Kuhlmann et al., 2019), as well as lessens the burden of deciding which families require screening. Given that 70% of the children in our study population did not have a documented injury prior to being diagnosed with maltreatment, a universal screening program may be the most effective child abuse and neglect prevention strategy that can be implemented in pediatric offices. However, most well-child visits last 20 minutes or less (Halfon et al., 2011) and pediatricians are increasingly asked to address more preventative health topics during these visits (Belamarich et al., 2006). These increasing time demands may make physicians reluctant to implement a universal child maltreatment risk factor screening program in their clinic. Additionally, physicians may be concerned about the sensitivity and specificity of child maltreatment screening tools and therefore may be hesitant to rely on them to identify cases of abuse and neglect (McTavish et al., 2020). In the absence of universal screening, physicians need to recognize children who are at an increased risk of experiencing harm or who have experienced injuries that may be concerning for abuse or neglect. Indeed, the American Academy of Pediatrics (2020) recommends that pediatricians engage in ongoing observation and assessment of family functioning during visits to prevent child maltreatment (Flaherty et al., 2010). Based on the results from our study, physicians should be particularly concerned about children less than 12 months of age who receive skeletal surveys, as well as those who are diagnosed with contusions, fractures, intracranial injuries, and retinal injuries. Even if the physician does not feel there is enough information to diagnose child maltreatment, these injuries can serve as an indicator of risk that prompts the physician to assess the family’s strengths and risk factors and refer the family to appropriate services in the community (Dubowitz et al., 2009, 2012).

### Limitations

Our results should be interpreted in the context of several limitations. First, we used administrative medical records (i.e., ICD-9-CM codes) to identify infants who experienced child abuse and neglect, which likely undercount the number of children who experience child maltreatment. Infants who experienced maltreatment but were not diagnosed as such would be excluded from our study population. It is possible that these infants experienced different injuries prior to being maltreated than the infants who were diagnosed with maltreatment. Second, we also used administrative medical records (i.e., ICD-9-CM codes) to identify prior injuries among children who experienced maltreatment, which may undercount the number of injuries experienced by infants. It is possible that pediatricians are less likely to document certain injuries, such as minor scratches and abrasions, in medical records. Third, we relied on Medicaid and CHIP claims data, limiting the generalizability of our findings beyond infants who are enrolled in these insurance programs. That said, given that over half of births in the United States are financed by Medicaid (Tanne, 2013), our findings are relevant to a large portion of infants in the United States. Fourth, our analyses were restricted to four states, limiting the generalizability of the findings to other states. However, we identified similar injuries among children who experience maltreatment as studies that used national data regarding pediatric hospital encounters (Lindberg et al., 2015; Puls et al., 2018), indicating the findings may still be relevant to all infants in the United States. Fifth, we did not include a control group in our analysis because our research questions were not predicated on comparing the prevalence of injuries among children who experience maltreatment to those who do not. Additionally, the potential for misclassification bias is high since many children who experience maltreatment are not diagnosed as such in their medical records. The absence of a control group precludes us from stating whether infants who are diagnosed with child maltreatment are at an increased risk of experiencing specific injuries compared to other children. That said, even without a control group, our study findings point to a high prevalence of concerning injuries among infants who are later diagnosed with child maltreatment. Such injuries in young children may function as a signal or early indicator of potential for harm to pediatricians and other healthcare providers, who can then intervene to prevent future harm to their patients.

## Conclusions

We identified relatively common injuries that occur prior to infants receiving a diagnosis for child maltreatment. Pediatricians should attend to infants in their practice who experience these injuries; refer their families to appropriate resources, such as home visiting and parent-training programs (Christian, 2015; Flaherty et al., 2010); and report families whose children have poorly explained injuries to CPS to reduce the likelihood of serious harm from child maltreatment.
